# Evaluation of an external foam column for *in situ* product removal in aerated surfactin production processes

**DOI:** 10.3389/fbioe.2023.1264787

**Published:** 2023-11-06

**Authors:** Chantal Treinen, Linda Claassen, Mareen Hoffmann, Lars Lilge, Marius Henkel, Rudolf Hausmann

**Affiliations:** ^1^ Department of Bioprocess Engineering (150k), Institute of Food Science and Biotechnology, University of Hohenheim, Stuttgart, Germany; ^2^ Cellular Agriculture, TUM School of Life Sciences, Technical University of Munich, Freising, Germany

**Keywords:** *in-situ* product removal, foam fractionation, surfactin, *Bacillus*, aerated fermentation processes, downstream processing

## Abstract

In *Bacillus* fermentation processes, severe foam formation may occur in aerated bioreactor systems caused by surface-active lipopeptides. Although they represent interesting compounds for industrial biotechnology, their property of foaming excessively during aeration may pose challenges for bioproduction. One option to turn this obstacle into an advantage is to apply foam fractionation and thus realize *in situ* product removal as an initial downstream step. Here we present and evaluate a method for integrated foam fractionation. A special feature of this setup is the external foam column that operates separately in terms of, e.g., aeration rates from the bioreactor system and allows recycling of cells and media. This provides additional control points in contrast to an internal foam column or a foam trap. To demonstrate the applicability of this method, the foam column was exemplarily operated during an aerated batch process using the surfactin-producing *Bacillus subtilis* strain JABs24. It was also investigated how the presence of lipopeptides and bacterial cells affected functionality. As expected, the major foam formation resulted in fermentation difficulties during aerated processes, partially resulting in reactor overflow. However, an overall robust performance of the foam fractionation could be demonstrated. A maximum surfactin concentration of 7.7 g/L in the foamate and enrichments of up to 4 were achieved. It was further observed that high lipopeptide enrichments were associated with low sampling flow rates of the foamate. This relation could be influenced by changing the operating parameters of the foam column. With the methodology presented here, an enrichment of biosurfactants with simultaneous retention of the production cells was possible. Since both process aeration and foam fractionation can be individually controlled and designed, this method offers the prospect of being transferred beyond aerated batch processes.

## 1 Introduction

**FIGURE 1:**
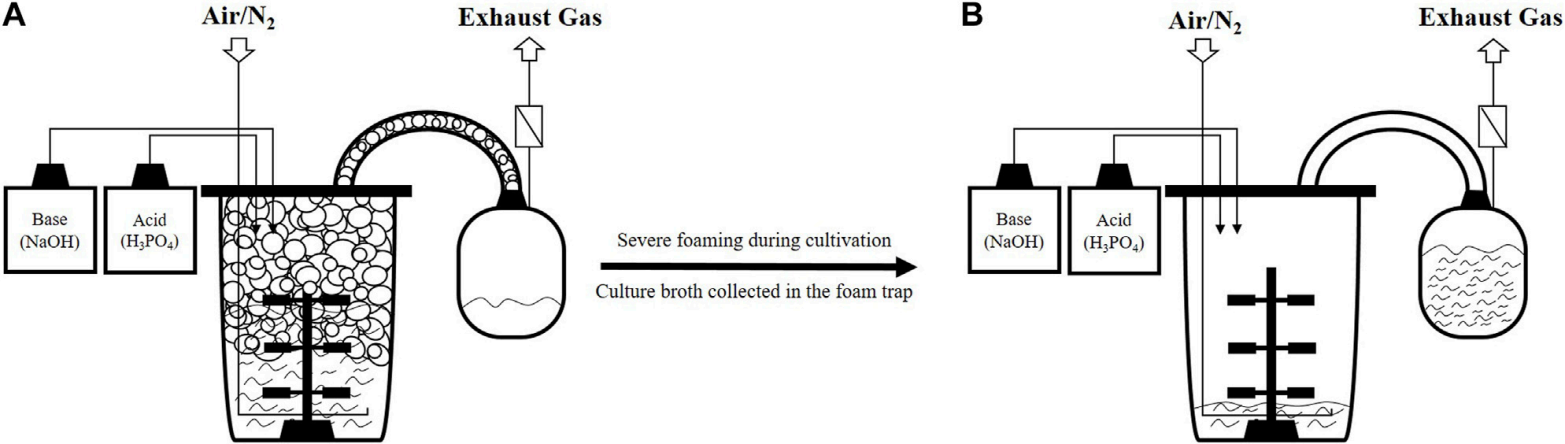
Schematic representation of overfoaming in aerated surfactin production processes using a foam trap. **(A)** Foam is building up in the headspace of the bioreactor, causing overflow; **(B)** Culture broth is mainly located in the foam trap, leaving the bioreactor almost empty as a result of severe foaming during the process [bioreactor figure adapted from Hoffmann et al. (2020)].

The fermentation process of *Bacillus* spp. to produce cyclic lipopeptides faces many challenges. Amongst them is excessive foam formation, especially during aerated cultivation (Coutte et al., 2017; Geissler et al., 2019a). Although foaming in bioreactor cultivations is generally present (Vardar-Sukan, 1998; Junker, 2007; St-Pierre Lemieux et al., 2019), this is severely increased for the production of microbial surfactants such as surfactin because the target product additionally has exceptional foaming capacities (Coutte et al., 2017). The foaming ability, alongside surface active properties (Arima et al., 1968) makes surfactin an attractive agent for various industries, including their use as detergents or emulsifiers (Coutte et al., 2017; Geissler et al., 2019a). Thereby, the isolation of production strains from food resources is of particular interest in this context to enable the application of lipopeptides, e.g., in the food sector (Akintayo et al., 2022). However, during surfactin production processes with uncontrolled foaming, foam formed in the headspace of the bioreactor can enter the exhaust line (Figure 1A) and lead to clogging of the exhaust filters (Vardar-Sukan, 1998). This can be associated with increased pressure in the bioreactor system and a severe loss in bioreactor volume due to overflowing (Vardar-Sukan, 1998; Davis et al., 2001) (Figure 1B). Another problem is that cells can be enclosed in the foam and therefore might be transferred with the culture broth out of the bioreactor system. In that case, cells as well as media can no longer be used for production (Vardar-Sukan, 1998; Coutte et al., 2017; Oraby et al., 2022). Therefore, some research studies have been aimed at developing surfactin production processes in which strong foam formation is circumvented. Examples include the use of a bubbleless membrane bioreactor (Coutte et al., 2010) or foam-free anaerobic cultivation (Willenbacher et al., 2015a; Hoffmann et al., 2020), further novel process strategies are summarized by Geissler et al. (2019a). However, the highest reported surfactin concentration of 26.4 g/L in laboratory scale was still reached during an aerated high-cell density fed-batch process by Klausmann et al. (2021). Intense foam destruction strategies had to be used in their process to cope with the strong foam formation, including mechanical and chemical methods. Additionally, to prevent a blockage of the filter system and collect overflowing culture broth, a foam trap (illustrated in Figure 1) can be connected downstream of the exhaust pipe as applied by Yeh et al. (2006) and Klausmann et al. (2021). The characteristic feature of the biosurfactant to accumulate at the gas-liquid interface can also be used as an advantage for process design (Coutte et al., 2017; Stevenson, 2019). Using *in situ* product removal (ISPR), the foam can continuously be collected, and the foaming capabilities of surfactin can be exploited (Rangarajan and Clarke, 2016). As the lipopeptide is enriched in the foamate, a first purification step in the downstream chain can be realized by using ISPR in the bioreactor process (Winterburn and Martin, 2012; Rangarajan and Clarke, 2015; Oraby et al., 2022). This would be advantageous insofar as the downstream process is known to be a high cost factor (Mukherjee et al., 2006; Winterburn and Martin, 2012). A recent review by Oraby et al. (2022) summarized foam fractionation in aerated stirred tank reactors, including coverage of numerous fractionation methods. They concluded that as of January 2021, foam fractionation was mainly applied for the production of biosurfactants, namely, in 74% of investigated cases. The vast majority of these studies use a method in which a foam trap is connected to the bioreactor systems. Thereby, the foam is collected via a pipe due to overflowing (Oraby et al., 2022). The earliest example of this method in surfactin production processes was conducted in Cooper et al. (1981). In comparison, a lower number of studies use a foam column, and external foam columns are used even less frequently (Oraby et al., 2022).

**FIGURE 2:**
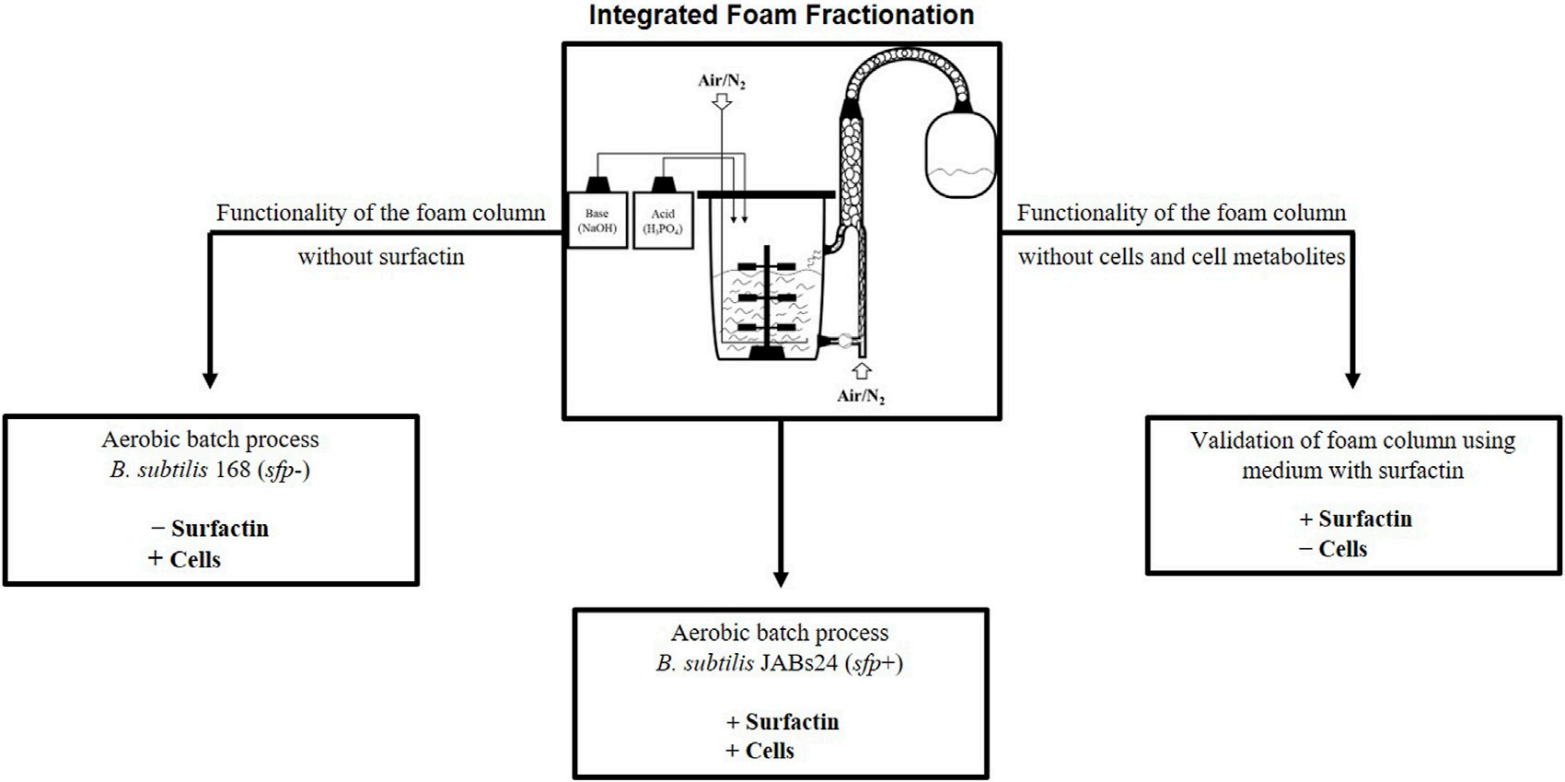
Integrated foam fractionation in aerated fermentation processes and experimental overview. A schematic representation of a bioreactor system with an external foam column is seen in the centre (bioreactor figure adapted from Hoffmann et al. (2020)).

**FIGURE 3:**
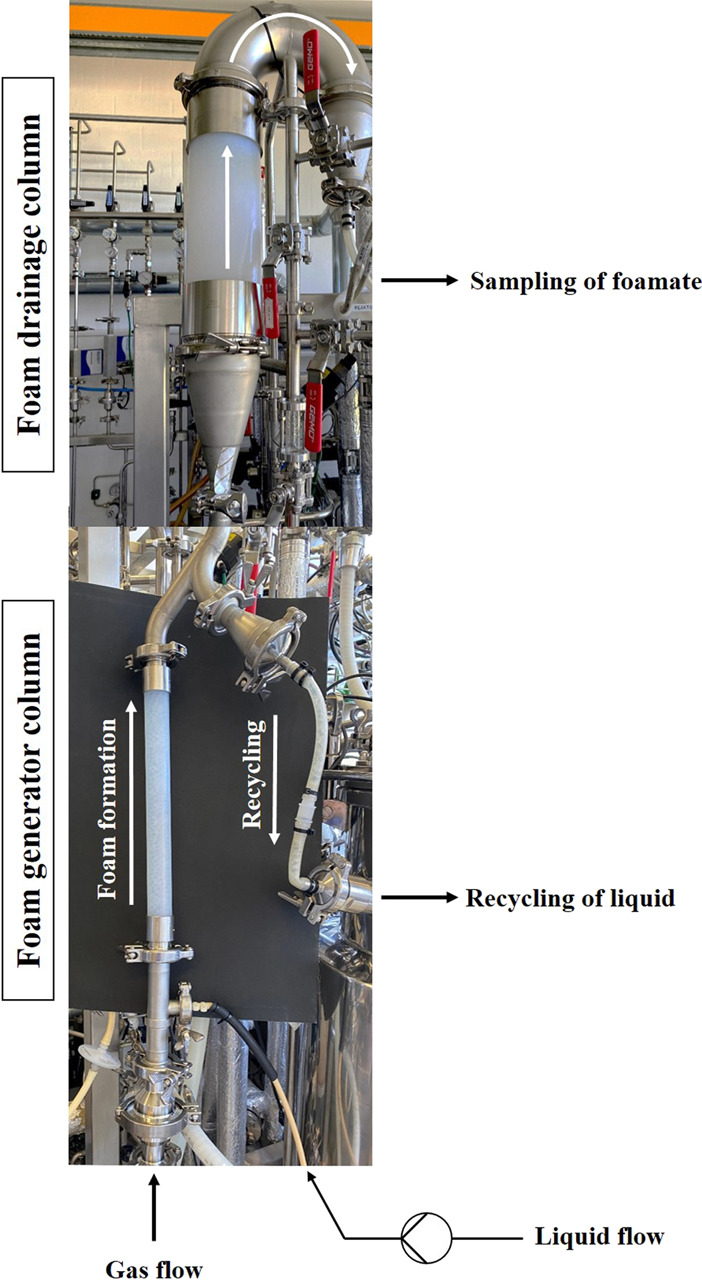
Image of the external foam column to illustrate the design and principle of operation. The foam column is connected to the bioreactor in this depiction. Due to the size, the lower part, which contains the foam generator column, and the upper drainage column were photographed separately. However, the two parts are connected at the point where the images were merged. The white arrows indicate the flow direction of the foam and the recirculated liquid.

In this study, an external foam column for integrated foam fractionation with a recirculation unit of the liquid is presented (Figures 2, 3), which can be categorized as “4b” according to the classification of Oraby et al. (2022). The number “4” refers to the method (integrated foam fractionation with an external column) and the letter “b” refers to the presence of a recirculation unit (Oraby et al., 2022). In the here applied method, the culture broth is sparged and brought to foaming in the foam column itself and is not collected via the headspace. In this way, the aeration of the foam column is not dependent on the aeration of the bioreactor system and is not used as a primary method for foam control, but rather as a tool for product enrichment. This is one of the highlights of the presented method, as it might potentially find application in foam-free and bubbleless fermentations. Since the culture broth is introduced into the foam column from below and then sparged, the system operates in the simple mode (Lemlich, 1968), and a pneumatic foam is created (Stevenson, 2019). A proof of principle is demonstrated using a standardized aerated batch process with the laboratory strain *Bacillus subtilis* JABs24. To test the influence of surfactin, cells and cell metabolites on the functionality of the foam column, two control experiments were performed. First, cell-free medium was examined with the addition of surfactin and then the non-surfactin producer *B. subtilis* 168 (*sfp* ^−^) was cultured as a negative control (Figure 2). In this way, the method can be evaluated and recommendations are made on the applicability of an external foam column with individual aeration using surfactin production in *Bacillus* as an example. Based on the faced challenges during the fermentation process, a need for smart control systems for future foam fractionation applications can be identified. Although external foam columns have generally been used for biosurfactant recovery (Blesken et al., 2020; Zheng et al., 2020), to the best of our knowledge this is the first time that this type of fractionation has been evaluated for surfactin production processes.

## 2 Materials and methods

### 2.1 Chemicals and standards

Chemicals of analytical grade were mainly received from Carl Roth GmbH and Co. KG (Karlsruhe, Germany), unless otherwise indicated. The reference substances for chromatographic analysis of surfactin (≥98% purity) and glucose (≥99.5% purity) were purchased from Sigma-Aldrich Laborchemikalien GmbH (Seelze, Germany). For functionality tests of the foam column, sodium surfactin (>90% purity) was received from Kaneka Corporation (Osaka, Japan).

### 2.2 Microorganism and strain maintenance

For bioreactor cultivation the non-surfactin producer *B. subtilis* 168 (DSMZ 23778, German Collection of Microorganisms and Cell Cultures GmbH, Braunschweig, Germany) was used in comparison to the surfactin producer *B. subtilis* JABs24 (Geissler et al., 2019b). The latter is based on strain 168 and has a corrected frameshift mutation in the *sfp* gene, thus allowing for surfactin production (Geissler et al., 2019b). For cryo-storage at −80°C, cells were preserved in lysogeny broth (LB) containing 15% (*v*/*v*) glycerol.

### 2.3 Media

As complex medium for preculture preparation, lysogeny broth (LB medium) was used, containing 10 g/L tryptone, 10 g/L NaCl and 5 g/L yeast extract (Bertani, 1951). A modified mineral salt medium (MSM) by Willenbacher et al. (2015b), based on Cooper et al. (1981), was used for bioreactor cultivations, containing a glucose concentration of 40 g/L. Media components were either sterilized by heat (15–20 min, 1 bar, 121°C) or by filtration (0.22 µm). To compensate for tryptophan auxotrophy of strain 168, tryptophan (50 μg/mL) was added respectively.

### 2.4 Bioreactor cultivation

Bioreactor cultivations were carried out as described in Treinen et al. (2021). Briefly, 20 kg aerobic batch processes were performed with 40 g/L glucose. The inoculation cultures were prepared in shake flasks as described in Treinen et al. (2021) with an incubation time of 16 h for preculture I and 16 h for preculture II. The bioreactor was inoculated with preculture II with the volume required to achieve an initial OD_600_ of 0.1 in the culture broth at t_0_ = 0 h. The fermentation process was then operated at 37°C and pH 7. The pH was controlled with 4 M NaOH and 4 M H_3_PO_4_. Online measurements of the pH were continuously performed using pH probes (EasyFerm Bio HB Arc 120, Hamilton Company, Nevada, United States). An aeration rate of 1.4 L/min process air (0.07 vvm) and an agitation of 300 rpm were initially set and then adjusted to maintain a pO_2_ of 20%. In case of difficulties during process operation, aeration and agitation were set manually. Online measurements of the pO_2_ [%], the aeration [L/min] and agitation [rpm] were recorded and are presented in the (Supplementary Figures S1–S5), also including further information on the individual process protocol. Online measurements of dissolved oxygen were continuously performed with respective probes (VisiFerm DO Arc 120, Hamilton Company, Nevada, United States). An external foam column was attached to allow foam fractionation, whereas reference processes did not include a foam column. Foam control was achieved as described previously (Klausmann et al., 2021; Treinen et al., 2021). Mainly a mechanical foam destruction using a foam centrifuge at 2,790 rpm was applied. For processes with an attached foam column, antifoam addition was avoided to maintain the foaming capacity. However, in cases of severe foaming which led to overflowing, antifoam (Xiameter^®^ AFE-1520; Dow Silicones Corporation, Midland, United States) had to be added with a syringe (max. 10 mL) to avoid a failure of the fermentation process. A 50 L foam trap was installed in the exhaust air line to avoid clogging of the filter system caused by a potential overflow, which simultaneously could be used as an alternative method of foam collection.

### 2.5 Sampling and sample analysis

Samples were taken at 3 h intervals starting at the beginning of cultivation at t_0_ = 0 h. During the night, the sampling interval was extended to 6 h. Cell density (OD_600_) was measured prior centrifugation using a spectrophotometer (Biochrom WPA CO8000, Biochrom Ltd., Cambridge, United Kingdom). Subsequently biomass was removed using centrifugation for 10 min at 4,816 *g* and 4°C (Heraeus X3R, Thermo Fisher Scientific GmbH, Braunschweig, Germany) and the resulting cell-free supernatant was preserved at −20°C. If necessary, centrifugation was performed twice to obtain a clear supernatant. From here, the production of surfactin as well as glucose and ammonia consumption during the course of cultivation were analyzed by measuring the respective concentration in the cell-free supernatant. Thereby surfactin and glucose measurements were conducted as described in Geissler et al. (2017) and Geissler et al. (2019b) using High-Performance Thin-Layer Chromatography (HPTLC) (CAMAG Chemie-Erzeugnisse und Adsorptionstechnik AG, Muttenz, Switzerland). Ammonia was determined photometrically with an assay kit, following the protocol of the manufacturer (Spectroquant^®^ Ammonium, Cat. No.: 114752, Merck KGaA, Darmstadt, Germany). Adjustments were made by reducing the volume to 5% in order to conduct the measurement in 96-well plates. Thereby a calibration range of 0.05–4 mg/L was considered. Standard as well as blank measurements were done regularly whilst performing the assay, if not stated otherwise.

### 2.6 External foam column

An external foam column was integrated into the process (Figure 3). A tube (d_i_ = 3.2 mm) was connected to a sampling valve at the bottom of the bioreactor. The culture broth was pumped (Masterflex^®^ P/S, Thermo Fisher Scientific GmbH, Braunschweig, Germany) from the bottom of the bioreactor into the foam column with a potential liquid flow of up to 25 mL/min. The foam fractionation unit was largely manufactured using stainless steel and consisted of two main parts. In the lower foam generator part of the column (H/D = 16, L = 400 mm, d_i_ = 25 mm; Vena^®^ View D25, Venair, Freiberg am Neckar, Germany), the culture broth was sparged, which was possible with either sterile process air or nitrogen through a sintered disk made of PTFE (RCT®-OHL-96, 10 µm pore size, Reichelt Chemietechnik GmbH + Co., Heidelberg, Germany). In this study mainly sterile process air similar to the bioreactor was used, unless otherwise stated. Gas flow rates of up to 10 L/min could potentially be applied. The foam could then rise into the upper drainage column (H/D = 3.9, L = 400 mm, d_i_ = 102 mm; Vena^®^ View D102, Venair, Freiberg am Neckar, Germany). Liquid and gas flow rates were specific for each experiment and are provided in more detail in the results section. Between the two column parts, a recirculation into the bioreactor was additionally installed. Cells and media components that were transferred into the foam column but not enriched in the foam could flow downwards by gravity, and thus be recycled back to the bioreactor. To prevent foam, that had formed in the headspace of the bioreactor, from passing into the foam column through the recirculation tube, a non-return valve was installed, if not specified otherwise (see Supplementary Table S3 for detailed process information). Foam that had accumulated in the drainage column could be transferred through an inverted U-shaped hose to a bottle for sampling. The weight of the sampled foamate was determined and if necessary the foam was liquefied with a drop of antifoam before further measurements. Samples obtained from the foam column were analysed for OD_600_ and surfactin concentration. The components of the foam column were mainly acquired from STAHLCON GmbH (Steinenbronn, Germany), VENAIR GmbH (Freiberg am Neckar, Germany), Reichelt Chemietechnik GmbH + Co. (Heidelberg, Germany) or kindly sponsored by VA GmbH Gesellschaft für Food Processing (Stuttgart, Germany).

#### 2.6.1 Functionality of the foam column without cells

To examine the foam column without the influence of cells and cell metabolites, 20 kg MSM (Willenbacher et al., 2015b) was added to the bioreactor. The medium was selected according to bioreactor procedures with the corresponding buffer concentration (4.29 × 10^−3^ M KH_2_PO_4_ and 5.71 × 10^−3^ M Na_2_HPO_4_ (Willenbacher et al., 2014)), but glucose addition was omitted. The medium was adjusted to pH 7 and the stirrer speed was set to 300 rpm and aeration to 1.4 L/min (0.07 vvm) at 37°C, thereby mimicking cultivation parameters of bioreactor cultivation. Surfactin (2 g/L) was added to the medium, as this concentration showed a good functionality of the foam column in a previous experiment (data not shown). In addition, strain JABs24 typically produces surfactin concentrations in the range of 1–3 g/L under comparable conditions (Geissler et al., 2019b; Hoffmann et al., 2021). The foam column was operated with process air using varying parameters, ranging from 7.5–15 mL/min liquid flow and 4.5–6 L/min gas flow. The parameters were chosen based on a previous experiment (data not shown). In addition, a foam trap was connected to the exhaust pipe to analyse the differences in enrichment between the two fractionation methods. For sampling with the foam trap, the foam centrifuge was switched off, resulting in an overflow shortly thereafter. Sampling was then possible via a bypass from the exhaust pipe. All samples from the medium, foam trap and foam column were taken in a comparable time-frame and analysed for surfactin concentration by HPTLC. In the case of the foam column, the duration of sampling and the weight of the foamate were additionally determined in order to calculate a flow rate of the foam.

#### 2.6.2 Functionality of the foam column without surfactin using *Bacillus subtilis* 168 (*sfp* ^−^)

To examine the functionality of the foam column without the presence of surfactin but in the presence of cells and cell metabolites, a bioreactor cultivation was performed, employing non-surfactin producer *B. subtilis* 168. On the second day of cultivation, when cells reached stationary phase, the foam column was operated with process air from approximately t ∼ 29.75 h to t ∼ 32.15 h. A liquid flow rate between 7.5–15 mL/min and a gas flow rate between 4.5–6 L/min for each set point was applied. The foam behavior in the column was noted and photographed. In contrast to the cultivation with surfactin producer *B. subtilis* JABs24, the cultivation with *B. subtilis* 168 was only carried out once with the attached foam column and without a reference process, as this experiment represents a negative control.

### 2.7 Data analysis and process parameters

Bioreactor cultivations employing strain *B. subtilis* JABs24 are displayed as biological duplicates. Additionally, technical replicates of offline parameters were typically carried out, resulting in at least a technical duplicate per sampling point. Plots were drafted using the scientific graphing analysis software Sigma Plot (Systat Software Inc., San Jose, United States). For glucose and ammonia depletion a curve fit was applied for visualization. Thereby, sigmoidal or logistic fitting curves with 4 parameters were implemented. To obtain the cell dry weight (CDW) in g/L, the optical density was divided by the correlation factor 3.3 ± 0.6 for strain JABs24 (Treinen et al., 2023) and 4.4 ± 0.1 for strain 168, which both were determined as described in Geissler et al. (2019b). Specific growth rate *µ* was calculated as described in Geissler et al. (2019b) using relative values. Thereby the highest growth rate determined is given as *µ*_max_, while *µ*_overall_ refers to the time span until the maximum biomass was reached. Surfactin and biomass enrichment were calculated for each foamate sampling point using Equations 1, 2 (Winterburn and Martin, 2012; Willenbacher et al., 2014).

$$\text{Surfactin enrichment}=\frac{c_{\text{Surfactin}\;\text{foamate}}}{c_{\text{Surfactin}\;\text{culture broth}}}$$ (1)

$$\text{Biomass enrichment}=\frac{c_{\text{Biomass}\;\text{foamate}}}{c_{\text{Biomass}\;\text{culture broth}}}$$ (2)

## 3 Results

### 3.1 Functionality of the foam column without cells

To determine an operation window of the foam column, MSM with different surfactin concentrations was tested by applying various operating parameters with sterile nitrogen used as gas flow. Based on these results, the following recommendations can be made for operating the foam column. In dependence on the estimated surfactin concentration achieved during cultivation, a liquid flow between 7.5–15 mL/min and a gas flow between 4.5–6 L/min provided good functionality of the foam column. In the next step, cell-free medium containing surfactin was used to compare the foam column with the foam trap, also analysing the influence of operating parameters. Thereby, the applied surfactin concentration was in range to that obtained in the performed cultivations with *B. subtilis* JABs24 (Table 3). The test was carried out starting with the lowest operating parameters. Samples were taken from the foam trap as well as the foam column (Table 1). The sampling with the foam column could take up to 1 h. The foamate collected reached m_foamate_ = 2.8–13.7 g with the foam column and m_foamate_ = 8.2–15.8 g with the foam trap. Thereby the surfactin concentration in the foamate averaged c_surfactin_ = 6.8 ± 2.5 g/L for the foam column and c_surfactin_ = 4.7 ± 1.0 g/L for the foam trap. However, the absolute amount of surfactin was similar for both methods, averaging 0.05 g surfactin in the foamate. The average enrichment with the foam trap was 3.3 ± 0.5 and 4.7 ± 1.4 with the foam column. Furthermore, a gas flow of 4.5 L/min resulted in overall higher surfactin enrichment, with 6.5-fold enrichment for a liquid flow of 7.5 mL/min and 5.8-fold enrichment for a liquid flow of 10 mL/min. However, these parameters also resulted in the lowest flow rate with only 0.07 g_foam_/min and 0.08 g_foam_/min. Vice versa, the highest flow rate of 0.23 g_foam_/min, which was achieved with the operating parameters of 7.5 mL/min liquid flow and 6 L/min gas flow, resulted in the lowest enrichment of 2.6. The measured surfactin concentration in the medium decreased with increasing test duration. From an initial surfactin concentration of 1.6 ± 0.0 g/L, only 75%, namely, 1.2 ± 0.1 g/L, remained in the medium. With an initial bioreactor volume of 20 kg, this led to a decrease in absolute surfactin values from 32.4 ± 0.2 g to 23.4 ± 1.8 g. Thereby the surfactin lost through sampling can be neglected as the weight of the discharged foamate was less than 100 g, which corresponded to a deduction of approximately 0.5 g surfactin (Table 1).

**TABLE 1:** Overview of results obtained by applying the foam column to the bioreactor filled with MSM and surfactin. To calculate the absolute amount of surfactin m_surfactin_, it was assumed that 1 g of foam equals 1 mL of foam. The absolute amount of surfactin for the medium was calculated with a volume of 20 L. Mean values of c_surfactin_ were used for the calculations of m_surfactin_ with standard deviations for the foamate typically ≤0.002 g.

Medium		Foam trap - foamate				Foam column - foamate						
c_surfactin_ [g/L]	m_surfactin_ [g]	c_surfactin_ [g/L]	Enrichment [-]	m_foamate_ [g]	m_surfactin_ [g]	Liquid flow [mL/min]	Gas flow [L/min]	Flow rate [g_foamate_/min]	c_surfactin_ [g/L]	Enrichment [-]	m_foamate_ [g]	m_surfactin_ [g]
1.6 ± 0.0	32.4 ± 0.2	6.3 ± 0.1	3.9	N/A	N/A	7.5	4.5	0.07	10.5 ± 0.1	6.5	4.2	0.04
1.5 ± 0.2	30.2 ± 3.4	5.4 ± 0.1	3.6	8.2	0.04	7.5	6	0.23	3.9 ± 0.0	2.6	13.7	0.05
1.5 ± 0.1	30.2 ± 1.4	3.4 ± 0.2	2.2	12.9	0.04	10	4.5	0.08	8.8 ± 0.1	5.8	2.8	0.02
1.4 ± 0.0	27.4 ± 0.6	4.0 ± 0.1	2.9	15.8	0.06	10	6	0.14	5.0 ± 0.0	3.7	8.4	0.04
1.2 ± 0.1	23.4 ± 1.8	4.6 ± 0.0	3.9	14.9	0.07	15	6	0.17	5.9 ± 0.1	5.1	10.8	0.06

### 3.2 Functionality of the foam column without surfactin

**FIGURE 4:**
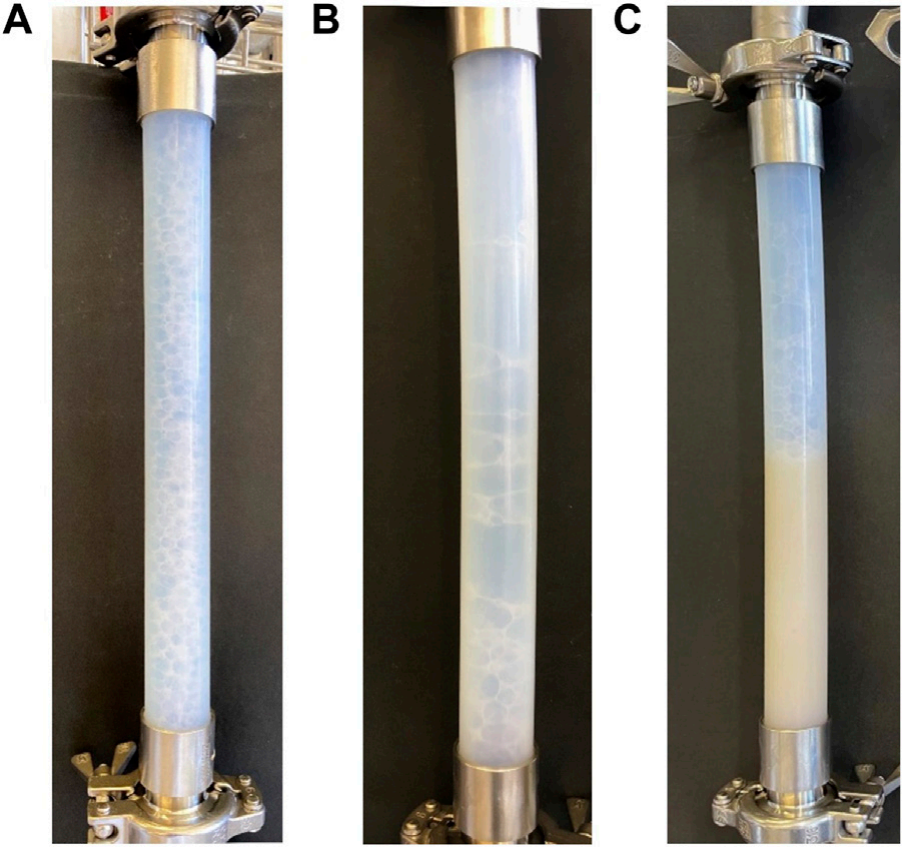
Exemplary images of foam behavior in the lower foam generator part of the foam column. Gas and liquid flow were co-current. **(A)** Small and stable foam bubbles which allowed for sampling. Photograph was taken during cultivation with surfactin producer *Bacillus subtilis* JABs24; **(B)** Foam bubbles were too large and burst quickly. Photograph was taken during cultivation with non-surfactin producer *Bacillus subtilis* 168; **(C)** Foaming was not possible, foam generator was filled with cultivation broth. Photograph was taken during cultivation with non-surfactin producer *Bacillus subtilis* 168.

As a negative control, non-surfactin producer *B. subtilis* 168 (*sfp* ^−^) was cultivated in the bioreactor. In this way, it could be analysed whether the functionality of the foam column was reliant on surfactin. Samples withdrawn from the bioreactor during cultivation were analysed for surfactin to control that the lipopeptide was not present. The HPTLC measurement confirmed that no surfactin was detectable with the method of choice and an application volume of 1 µL. During cultivation, foam formation was visible in the bioreactor system. When reaching stationary phase, the external foam column was operated using various parameters (Table 2). However, sampling was not possible with any of the tested parameters. In general, it was observed that although bubbles formed in the foam generator part of the foam column, the stability seemed not sufficient for the foam to rise in the drainage column. Most of the foam collapsed and caused the foam generator column to fill up with liquid. Also, the broth accumulated in the recirculation tube and was recycled to the bioreactor. Exemplary images of foam behavior in the column are presented in Figure 4. Here it can be seen that during cultivation with surfactin producer JABs24 small, almost evenly distributed bubbles were formed (Figure 4A). In comparison bubble size varied and was increased when cultivating non-surfactin producer 168 (Figure 4B). Additionally, the foam generator part filled up with liquid (Figure 4C). The last two observations were occasionally also found during cultivations with JABs24 in dependence of the chosen operation parameters and point of time during cultivation.

**TABLE 2:** Overview of the various operating parameters of the foam column and the associated foam behavior using *Bacillus subtilis* 168.

Liquid flow [mL/min]	Gas flow [L/min]	Sampling/comment
7.5	4.5	Bubbles too big and burst quickly, accumulation of foam in the recirculation tube, no foam accumulated in the foam generator
7.5	6	Bubbles too big and burst quickly, accumulation of foam in the recirculation tube, no foam accumulated in the foam generator
10	4.5	Bubbles not stable, foam generator column filled with culture broth
10	6	Bubbles too big and burst quickly, accumulation of foam in the recirculation tube
15	4.5	Bubbles not stable, foam generator column filled with culture broth
15	6	Small bubbles, accumulation of foam in the recirculation tube, foam generator column filled with culture broth

### 3.3 Cultivation of *Bacillus subtilis* strain with integrated foam fractionation

**FIGURE 5:**
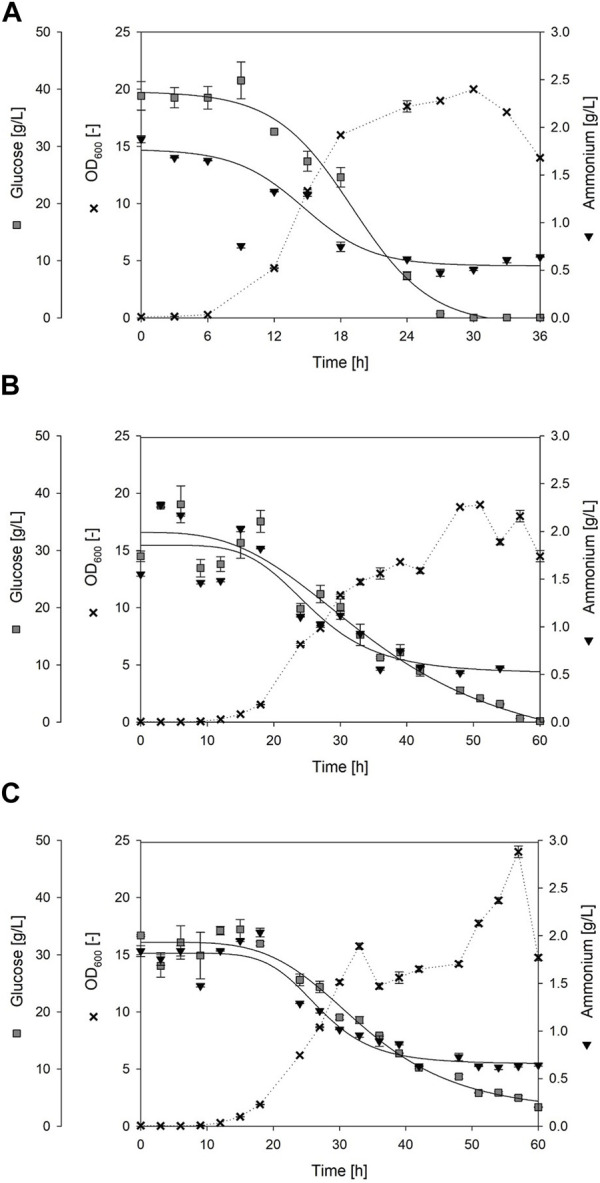
Time course of bioreactor batch cultivations using *Bacillus* spp. Exemplary processes are shown, each representing one biological replicate with **(A)** strain *Bacillus subtilis* 168 until t = 36 h; **(B)** *Bacillus subtilis* JABs24, Replicate 1 until t = 60 h; **(C)** *Bacillus subtilis* JABs24 with integrated foam fractionation (ISPR), Replicate 1 until t = 60 h. Given are the cell growth as OD_600_ (black cross), the consumption of the carbon source glucose (gray square) and the consumption of the nitrogen source ammonium (black triangle) over the cultivation time. Solid lines indicate a dynamic curve fit that is either sigmoidal or logistic with 4 parameters. Curve fit of ammonia for *Bacillus subtilis* 168 does not include time-point t = 9 h. The dashed lines, however, do not represent a fit and are only integrated for simplified visualization.

Batch cultivations were performed with and without an external foam column to evaluate integrated foam fractionation in aerated surfactin production processes. An overview and detailed process information of all performed batch cultivations of *B. subtilis* JABs24 with the external foam column is presented in Supplementary Table S3, also including failed experimental runs. Figure 5 shows exemplary time-courses of the bioreactor cultivations up to t = 60 h, also featuring the negative control using non-surfactin producer *B. subtilis* 168 up to t = 36 h. Corresponding online measurements are illustrated in the Supplementary Material (*B. subtilis* 168, Figure 5A and Supplementary Figure S5; *B. subtilis* JABs24 Reference process; Figure 5B and Supplementary Figure S1; *B. subtilis* JABs24 Foam column process; Figure 5C and Supplementary Figure S3). In terms of cell growth and maximum optical densities, a similar trend was observed for all three fermentation processes. Differences mainly occurred in the duration of the lag phase and the overall cultivation time. In the reference process with strain JABs24, the highest OD_600_ value of 19.0 ± 0.0 was registered after 51 h with a maximum growth rate of *µ*_max_ = 0.40 1/h (Table 3). For the proof of principle with an attached foam column, the highest OD_600_ value of 24 ± 0.5 was reached after 57 h with maximum growth rate of *µ*_max_ = 0.45 1/h. In comparison, the non-surfactin producer *B. subtilis* 168 reached the maximum OD_600_ after 30 h with 20.0 ± 0.0 and a growth rate of 0.46 1/h. After glucose depletion, a reduction in biomass could be detected in all experimental approaches. However, in the foam column process a residue of 3.3 ± 0.1 g/L glucose remained. In terms of the nitrogen source, residual concentrations of around 0.6 g/L were measured in the culture broth.

**TABLE 3:** Overview of process parameters for exemplary bioreactor cultivations with *Bacillus subtilis* JABs24. The foam column was operated with a liquid flow of 15–20 mL/min and a gas flow of 3 L/min. Further information on the individual foam samples are provided in Supplementary Tables S1, S2.

Parameter	Foam column process Replicate 1		Foam column process Replicate 2	
X_max_ [g/L] - Culture broth	7.4 ± 0.2	57 h	6.3 ± 0.2	30 h
P_max_ [g/L] - Culture broth	1.9 ± 0.0	51 h	2.6 ± 0.0	48 h
X_max_ [g/L] - Foamate	2.7 ± 0.0	51 h	5.7 ± 0.2	30 h
P_max_ [g/L] - Foamate	6.4 ± 0.3	51 h	7.7 ± 0.4	51 h
X Enrichment_max_	0.5	60 h	2.4	54 h
P Enrichment_max_	4.0 3.3*	24 h 51 h	3.3	51 h
X Enrichment_mean_	0.4 ± 0.1		1.8 ± 0.6	
P Enrichment_mean_	2.7 ± 0.5		2.3 ± 0.7	
*µ*_max_ [1/h]	0.45		0.63	
*µ*_overall_ [1/h] at X_max_	0.11		0.20	

Parameter	Reference process Replicate 1		Reference process Replicate 2	
X_max_ [g/L] - Culture broth	5.8 ± 0.0	51 h	5.7 ± 0.2	24 h
P_max_ [g/L] - Culture broth	3.6 ± 0.1	33 h	3.0 ± 0.0	54 h
*µ*_max_ [1/h]	0.40		0.52	
*µ*_overall_ [1/h] at X_max_	0.12		0.24	

*Without initial high-point at t = 24 h.

X = Biomass (CDW, cell dry weight).

P = Product (surfactin).

### 3.4 Overfoaming and regulation challenges during bioproduction

Severe problems with overfoaming and media loss were observed for most cultivation runs with strain JABs24 (Supplementary Table S3). For example, a reduction from an initial 19.5 kg at t_start_ to 12.0 kg at t_end_ was observed in replicate 2 of the foam column process (Supplementary Figure S4B). Thereby an interval-like decrease in reactor volume was seen with the biggest drop at t ∼ 23 h. Within a timeframe of approximately 10 min, only 13.6 kg of an initial 18.3 kg remained in the bioreactor. This means that about a quarter of the reactor volume, namely, 4.8 kg (26.2%) was lost due to uncontrollable foaming in a short time interval. In severe cases, overfoaming potentially led to complete failure and premature termination of the experiment. In one of these fermentations 11.3 kg, responding to 55.9% of the initial volume were lost within only 20 min and in another case <3 kg of culture broth remained in the bioreactor, which also resulted in the probes no longer being covered properly (Supplementary Table S3). Whereas the processes employing the external foam column could not or only slightly be regulated using chemical antifoam agent, the application of such was possible in the reference process in addition to the foam centrifuge. Nonetheless an overfoaming was still observed for one of the reference processes, namely, for replicate 2 (Supplementary Figure S2B). Within a time-frame of 2 h (between t ∼ 22:35 h and t ∼ 24:35 h) a reduction of the reactor volume from an initial 20.1 kg to a volume of 15.9 kg was observed. This resulted in a media loss of 4.3 kg (21.3%). In general, overfoaming was observed when agitation and sometimes also aeration were increased with stirrer speeds up to a maximum level of >800 rpm to maintain the set pO_2_ level. It was also found that foam from the headspace could potentially be forced through the recirculation tube. Therefore, a non-return valve was installed in the course of experimental runs to improve this (see Supplementary Table S3 for detailed process information). Another challenge during the bioreactor processes was maintaining a continuous pO_2_ level, as the regulation occasionally failed to operate (see individual processes in the Supplementary Material for more detailed information on process performance). Among other factors, the addressed challenges during bioproduction made it difficult to achieve reproducibility of the process performance.

### 3.5 Surfactin and biomass enrichment with integrated foam fractionation

**FIGURE 6:**
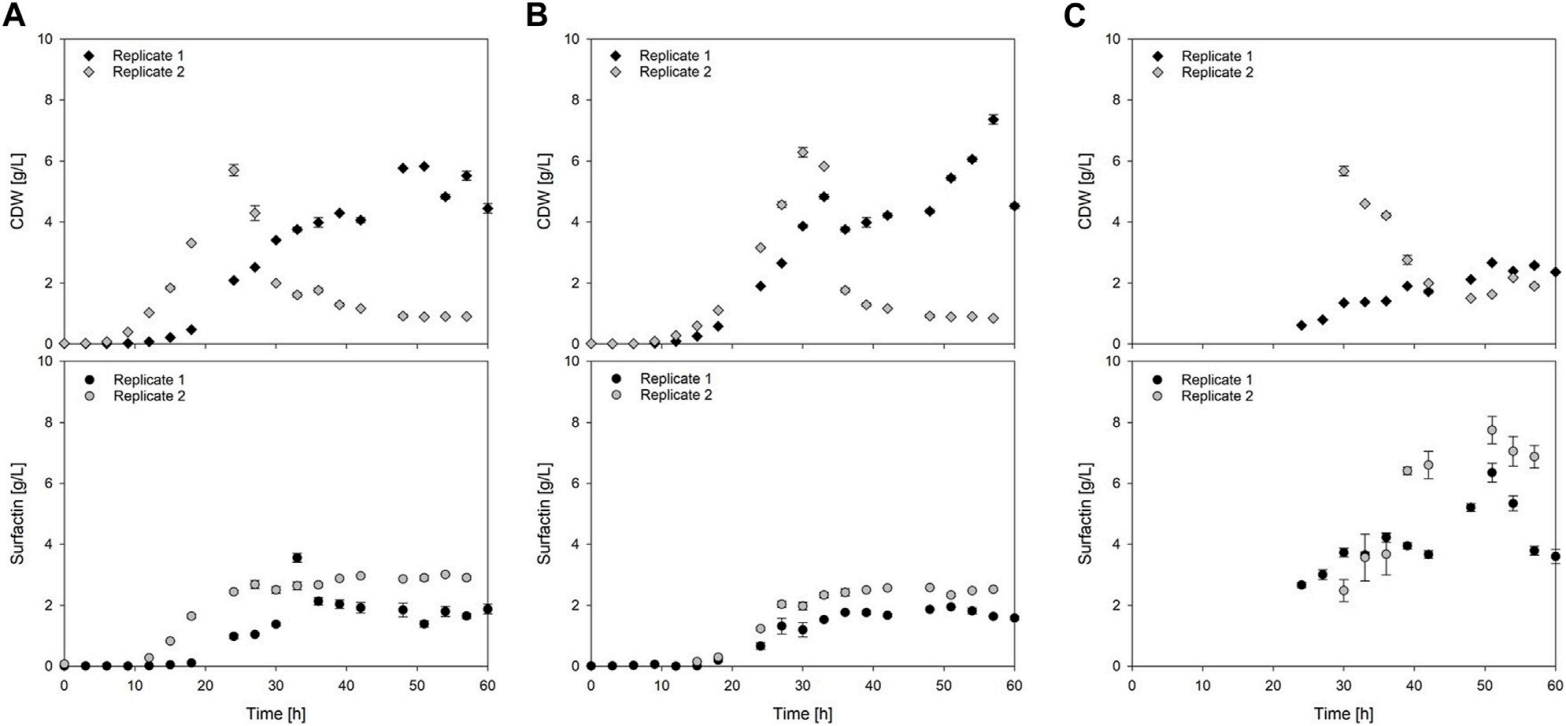
Time-course of cell dry weight (CDW) and surfactin production during exemplary batch cultivations. **(A)** Reference process with *Bacillus subtilis* JABs24 without foam fractionation; **(B,C)** Foam column process as proof of principle with *Bacillus subtilis* JABs24 and integrated foam fractionation (ISPR). For the latter, **(B)** concentrations in the culture broth; **(C)** concentrations in the foamate.

**FIGURE 7:**
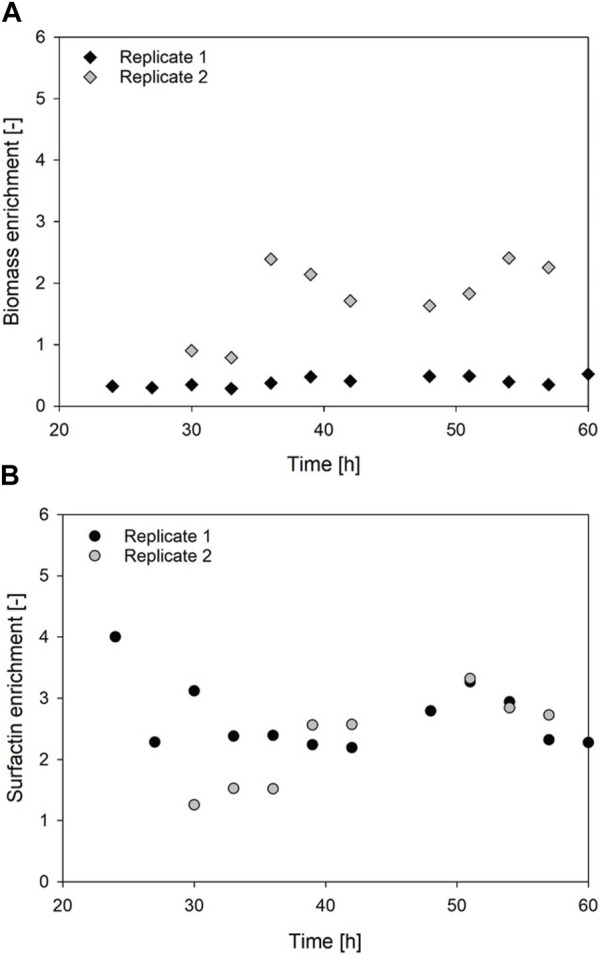
Time-course of biomass and surfactin enrichment for exemplary batch cultivations. **(A)** Biomass enrichment; **(B)** Surfactin enrichment. Represented are the calculated values for the proof of principle process, employing *Bacillus subtilis* JABs24 with integrated foam fractionation (ISPR).

The challenge to reach reproducibility, that has been seen for the reactor volume and the dissolved oxygen pO_2,_ has also been noticeable in the overall biomass time-course as exemplary visualized in Figures 6A, B. However, a trend could still be observed as the reference processes reached similar CDW_max_ of 5.8 ± 0.0 g/L and 5.7 ± 0.2 g/L but at different time points during cultivation (Table 3). Replicate 2 reached the maximum biomass already after 24 h with a slightly higher specific growth rate of *µ*_overall_ = 0.24 1/h, whereas replicate 1 reached its maximum after 51 h with *µ*_overall_ = 0.12 1/h. A similar trend was observed for the foam column process. Again the highest biomass was reached time-delayed although showing a similar trend up until the end of the exponential phase. Replicate 1 reached its maximum of 7.4 ± 0.2 g/L after 57 h with a specific growth rate of *µ*_overall_ = 0.11 1/h. Replicate 2 however reached the highest biomass already after 30 h with a CDW_max_ of 6.3 ± 0.2 g/L and a slightly increased growth rate of *µ*_overall_ = 0.20 1/h (Table 3). Interestingly the biomass formation in the foam column process was increased in both cases compared to the reference processes. The highest overall biomass in the foamate was detected for replicate 2 with 5.7 ± 0.2 g/L after 30 h (Table 3). The highest overall biomass enrichment of 2.4 was observed for the same replicate after 54 h (Table 3; Figure 7A). In general, the mean values of biomass enrichment ranged from 0.4 ± 0.1 to 1.8 ± 0.6 between replicates (Table 3). Despite observed variations in bacterial growth, the overall trend for surfactin production over time was comparable (Figure 6). Maximum product concentrations of 3.6 ± 0.1 g/L and 3.0 ± 0.0 g/L surfactin were reached during reference processes. When applying the foam column, surfactin concentrations in the culture broth were lower compared to the reference processes. Here surfactin levels of P_max_ = 1.9 ± 0.0 g/L and P_max_ = 2.6 ± 0.0 g/L were reached after 51 h and 48 h respectively (Table 3). Surfactin concentration in the foamate was increased and levels of 6.4 ± 0.3 g/L and 7.7 ± 0.4 g/L were determined towards the end of the cultivation at t = 51 h. Although variations in bacterial growth and hence in maximum surfactin concentrations were observed between the replicates, a different picture emerged for surfactin enrichment (Figure 7B). Despite an initial peak for replicate 1 at the beginning of foam collection (4.0-fold enrichment after 24 h), the highest enrichments obtained were generally similar with a maximum enrichment of 3.3 after 51 h (Table 3). From about 40 h of cultivation, enrichment values have converged and settled between 2–3. Across the replicates, the mean enrichments were 2.7 ± 0.5 for replicate 1 and 2.3 ± 0.7 for replicate 2. Additional information on the individual sampling points with regard to surfactin enrichments, foam column parameters and flow rates of the foamate is provided in Supplementary Tables S1, S2. Interestingly during the bioreactor cultivations, the parameters of the foam column were adjusted depending on the foam formation and the process stage and were not limited to the previously defined operation window. The liquid flow was generally between 10–20 mL/min and the gas flow was between 3–9 L/min (Supplementary Table S3). This resulted in flow rates of the foamate between 0.03 and 1.91 g_foamate_/min (Supplementary Tables S1, S2).

## 4 Discussion

### 4.1 Partial transition of surfactin into the foam

An experiment was conducted in which the bioreactor was filled up with 20 kg MSM and supplemented with surfactin. At the end of the experiment, it was observed that the measured concentration in the medium decreased from 1.6 ± 0.0 g/L surfactin to an amount of 1.2 ± 0.1 g/L, which referred to 75% of the initial measured concentration. Since foam fractionation is intended to remove surfactin from the process, it seems logical at first that the concentration in the bioreactor would decrease. However, in this experiment, the absolute amount of discharged surfactin only accounted to 0.5 g. Therefore, the observation rather indicated that the surfactin distributed to a large extent in the foam which was built in the headspace of the bioreactor. This would also explain why the measured concentration in the medium was 1.6 g/L, although 2 g/L were initially added. The results and the hypothesis are in line with literature findings, in which it was stated that the largest proportion of surfactin was accumulated in the foam samples (Cooper et al., 1981; Davis et al., 2001; Coutte et al., 2010). To put the observation in numbers, Coutte et al. (2010) described that in one of their experiments, 714 mg surfactin were found in the foam whereas only 60 mg of surfactin were measured in the culture broth. This is an important factor to consider, as samples during bioreactor cultivation are often taken only from the culture broth to analyze titer and yield of the produced biosurfactant. In that case, the amount of foam in the headspace alongside the concentration of the biosurfactant in the foam is neglected, suggesting even higher titers and yields than described in the literature.

### 4.2 Surfactin enrichment with different fractionation methods and influence of recirculation

Using the surfactin enriched MSM in the bioreactor, samples were withdrawn with a foam trap as well as a foam column for a direct comparison. It was observed that the average surfactin enrichment achieved with the foam column (4.7 ± 1.4) surpassed the surfactin enrichment using the foam trap (3.3 ± 0.7). This might be explained by the so-called drainage effect. As the foam flows upward in the foam column, or builds up in the headspace, culture broth is also being held in the foam. Due to gravity, the liquid part can flow down, which results in a dry and enriched foamate (Burghoff, 2012; Winterburn and Martin, 2012; Oraby et al., 2022). This allows larger parts of unwanted substances such as cells and medium to be rinsed down, which further increases the enrichment in the foamate (Burghoff, 2012; Oraby et al., 2022). The set-up of the foam column used in this study additionally had a recirculation unit in which the liquid part could be directed back to the bioreactor. This recycling step was not possible with the here applied foam trap which might explain the overall lower enrichments achieved compared to the foam column. Interestingly, it has been reported in the literature that the recycling of collapsed foam or foaming of cells has a positive impact on surfactin production as well as cellular growth (Atwa et al., 2013; Alonso and Martin, 2016). In addition, the removal of the lipopeptide was suggested to be advantageous for production efficiency (Cooper et al., 1981), and that high surfactin concentrations also have a negative impact on bacterial growth (Lilge et al., 2022). In this study higher biomass titers were achieved in the foam column process compared to the reference process, which is in accordance with the literature findings. However, surfactin titers in the culture broth were not increased when introducing the foam column. In fact, concentrations that were in range of typically produced surfactin titers by *B. subtilis* JABs24 (between 1–3 g/L) were observed (Geissler et al., 2019b; Hoffmann et al., 2021). This indicates that the positive effect of foaming described above was not clearly recognizable at first glance. Reasons for this might have been due to the low flow rates of the foamate, as maybe not as much foam and/or cells have been removed and recycled. Moreover, cells were subjected to foaming in both process scenarios. In order to understand the findings and correlations even better, further investigations also regarding production efficiency can be considered in follow-up studies.

### 4.3 Interrelation of flow rates and surfactin enrichment

In principle, the flow rates of the foamate were in range to the flow rates described in the literature (Davis et al., 2001; Chen et al., 2006; Santos da Silva et al., 2015). However, despite sufficient surfactin enrichment, the flow rates were rather low with mainly less than 1 g_foamate_/min being collected. It was observed that a lower aeration also resulted in a lower flow rate as it took longer for the foam to travel through the foam column. Simultaneously it was observed that the sample with the lowest flow rate also had the highest enrichment. This was most likely due to an increased time for drainage (view chapter 4.2) as Zhang et al. (2015) described that a prolonged retention time in the column positively affects the foam dryness. This observation was not unexpected, as Stevenson (2019) pointed out the great importance of aeration on the foam fractionation performance, describing that low aeration leads to high enrichments at a low flow rate of the foam. A special feature of the applied method in this study is, that the foam column and especially the aeration can be operated independently of the bioreactor system. The influence of aeration and agitation on foaming as well as enrichment was also examined in studies by Santos da Silva et al. (2015) and Davis et al. (2001). In the described examples the aeration and agitation were monitored however in the bioreactor itself, as the foam column was attached to the headspace of the bioreactor. Again, high agitation and/or aeration was found to increase foam formation, which was associated with a higher flow rate of the foamate, but at the same time, lower enrichment of surfactin (Davis et al., 2001; Santos da Silva et al., 2015).

### 4.4 Influence of surfactin and cells on functionality of the foam column

Although foam formation occurred during cultivation of *B. subtilis* 168 in the bioreactor, it appeared that surfactin was required for the functionality of the foam column. Foam formation itself in aerated fermentation processes is not unusual (Vardar-Sukan, 1998), however the foam was not stable enough to rise in the foam column. This underlined that the presence of surfactin was required to operate the column in this process. This observation was as expected, since surfactin is a surface-active compound (Arima et al., 1968) and as such is able to stabilize the foam (Burghoff, 2012; Stevenson, 2019). Due to this feature, Burghoff (2012) even described foam fractionation as a method to separate surface-active compounds from other metabolites of the culture broth. Additionally, in a study by Chen et al. (2006) it was observed that a minimum of 10 mg/L of surfactin was required to achieve foaming.

However, not only surfactin had an influence on foam stability, but also the presence of cells and cell metabolites. Davis et al. (2001) described that in a cell-free operation, the foaming capacity was lowered and hypothesized that cells have a positive effect on foaming. Additionally, it was also determined, that cell-free samples had a higher enrichment (Davis et al., 2001), which was in line with here obtained results. The average enrichment in cell-free medium (4.7 ± 1.4) was higher compared to the average enrichment during cultivation (2.5 ± 0.6). Junker (2007) summarized in their review various factors that influence foam stability during cultivation, amongst them were cells and cell metabolites, medium components, and bacterial growth. Another interesting fact to consider is the natural ability of *Bacillus* species to secrete proteins and enzymes (van Dijl and Hecker, 2013). Foam fractionation is not limited to the separation of biosurfactants, but is also applied for proteins (Stevenson, 2019; Oraby et al., 2022) which, among other metabolites, can have a positive effect on foam stability (St-Pierre Lemieux et al., 2019). When using foam fractionation in the negative control *B. subtilis* 168, the foam column did not work without the surface-active lipopeptide being present. However, protein quantification or further protein investigation was not considered in the experiments but could be an interesting element for future works, especially when applying strains that produce large amounts of protein. In this context it would then also be interesting to conduct a proteomic analysis of the cell-free supernatant to gain clarity on the extracellular proteins present during fermentation.

### 4.5 Integrated froam fractionation in surfactin production processes

The mean biomass enrichment in the foam varied between 0.4 ± 0.1 and 1.8 ± 0.6, which was in similar range of a study by Willenbacher et al. (2014), with a mean bacterial enrichment of <0.2 to 1.6. Alongside the bacterial growth, surfactin production also showed deviations, though maintaining a similar trend. For Replicate 1 an initial highpoint of a 4.0-fold enrichment was measured at the beginning of sampling. An initially high surfactin enrichment was also observed by Willenbacher et al. (2014) and Santos da Silva et al. (2015). This might also be caused by the previously discussed drainage effect (see chapter 4.2) as it took some time for the foam column to run before sampling was possible. Therefore, the first sample might have been more dry and therefore more enriched (Burghoff, 2012). This characteristic high point at the beginning of sampling might have been missed in the second replicate. Despite difficulties in reproducibility and a large number of influencing parameters, both the average and maximum surfactin enrichment were in a comparable range between 1.0–4.0 when looking at the overall performed experiments (Supplementary Table S3). However, the achieved surfactin enrichments in this process were generally lower compared to other studies. For example, Davis et al. (2001) and Chen et al. (2006) achieved enrichments of >50. Results by Santos da Silva et al. (2015) were more comparable with enrichments of 1.4–7.4 depending on aeration and agitation rates. However, the highest enrichment obtained in their study (28.7) was still considerably higher than the values obtained here. These differences in enrichment could have been due to a number of reasons. First, it should be noted that different cultivation and foam fractionation methods have been applied, as well as different bacterial strains, which makes a direct comparison difficult. Especially the influence of aeration and agitation rates during the process have been proven to have an influence on the surfactin concentration and enrichment (Davis et al., 2001; Santos da Silva et al., 2015) as discussed in chapter 4.3. The design of the foam column also has an influence on the drainage effect and thus on the enrichment of the surfactant (Zhang et al., 2015; Oraby et al., 2022). As shown by Willenbacher et al. (2014), the choice of the bacterial strain should also not be neglected. They found a discrepancy as surfactin enrichment was ranging from 12.7 ± 1.0 (DSM 3258) to 161.1 ± 6.0 (DSM 1090) in dependence of the used strain. These observations suggest that before applying ISPR for lipopeptide production, not only the choice of foam fractionation method, but also the choice of fermentation process and bacterial strain should be carefully evaluated. Oraby et al. (2022) came to similar conclusions in their literature review by pointing out that there is no standardized foam fractionation method and emphasizing the need for individual process design. This indicates that the research results obtained in this study and the conclusions from the literature review are complementary and supportive of each other, also with regard to the difficulties encountered during foam fractionation (Oraby et al., 2022).

### 4.6 Evaluation of foam fractionation in aerated surfactin production and need for control system

Although a mechanical foam disruption technique was used, the foaming was difficult to control in the bioreactor vessel. Overfoaming occurred regularly during cultivations with surfactin producer *B. subtilis* JABs24, also leading to failure of the experiments (Supplementary Table S3). An application of chemical antifoam was problematic, as foaming itself is essential to enable sampling. It has to be noted that even in the antifoam-controlled reference processes, overfoaming occurred as both the mechanical and the chemical foam disruption were not sufficient. Intense loss in culture medium was also observed during foam fractionation processes in Davis et al. (2001) and Santos da Silva et al. (2015). The deduction of culture broth in other studies could only be contained by lowering the aeration and agitation rates (Santos da Silva et al., 2015) or compensated by using two subsequent vessels to collect the overflowing liquid with a recycling unit to the bioreactor (Gong et al., 2009). Furthermore, it was seen that the overfoaming sometimes happened in a wave-like manner (Supplementary Figure S4B). This suggests that a collection of foam during cultivation over a foam trap seems to be challenging as continuous foam removal can be difficult to reach. This was already put forward by Rangarajan and Clarke (2015) who included the “*mainte-nance of uniform foaming characteristics and foam stability*” (Rangarajan and Clarke, 2015) as a challenge for foam fractionation. However, as reviewed by Oraby et al. (2022) the use of a foam trap is still one of the favoured methods for foam fractionation and is used in more than 50% of the applications studied, and when the products were biosurfactants, foam traps were used in as many as two-thirds of the cases. Besides the overfoaming, sensitivity towards fluctuations in pO_2_ regulation and difficulty in maintaining a steady measurement were observed, possibly explaining differences in bacterial growth and surfactin production throughout the biological replicates. The extremely non-stationary behavior during fermentation due to strong foam formation with corresponding inhomogeneity might have hampered the measurements of the pO_2_ probes (Vardar-Sukan, 1998; St-Pierre Lemieux et al., 2019), apart from potential technical performance problems. However, a stable pO_2_ regulation is crucial as we previously discussed that aeration and agitation directly influence the foam behavior and enrichment (view chapter 4.3). Therefore, the regulation of it should be reliable and robust. These challenges highlight the need for a better control system that continuously measures foam formation and not only monitors and regulates pO_2_, but is also able to adjust to process dynamics. This seems plausible when looking at how the foam is built in the foam column. As described by Stevenson (2019), there are three phases: i) the “*liquid pool*” or “*pulp phase*”, ii) the “*foam phase*” where the foam flows up the column and iii) the “*collection zone*” in which the foam can be sampled (Stevenson, 2019). If no or too little surfactin was present or if parameters were not adjusted accordingly, it was observed in this study that the phases were not in equilibrium to allow continuous sampling (Figure 4), for example, the liquid pool was too extensive (Figure 4C). The way the foam is built up in the foam generator column, which is decoupled from the bioreactor system, displays one of the highlights of the presented method and is a decisive distinction to a foam column that is directly connected to the bioreactor headspace. However, foam control was not achievable with the here applied set-up as the primary method, so additional measures had to be taken. Follow-up studies could therefore consider the application of the foam column in processes with minimal or no foam formation so that the advantages can be fully exploited. But also, the bubble size distribution in the foam is important for the success of foam fractionation and it is difficult to be measured and to be reproduced (Stevenson, 2019). Although its concrete influence is not fully elucidated as of yet, it is known that the bubble size (distribution) determines the dryness and the flow rate of the foamate and thus the overall perfomance (Stevenson, 2019). This is another reason why antifoam can hardly be used because it affects the bubble size and leads to heterogeneous bubble distribution (Al-Masry et al., 2006). To control these difficulties and to achieve an optimal ratio between the enrichment and the flow rate of the foamate, dynamic monitoring in the sense of a model-based process control (Oraby et al., 2022) or a specific sensor system would be of interest to address the discussed obstacles. Current advances on modelling foam fractionation focus on the broad parameters influencing foam fractionation. For example, a quasistatic model for foam fractionation was developed by Grassia (2023) to better understand the influences of operating conditions on the fractionation process and performance. In addition, García-Figueroa et al. (2023) worked on a model to also consider the biochemical properties and adsorption of the product as an influence on the separation. Another recent attempt was made by Keshavarzi et al. (2022), modeling BSA recovery by froth flotation with an emphasis on bubble size distribution as well as foam stability. These approaches underline the complexity of foam fractionation processes and proof that model implementation can be very extensive.

## 5 Conclusion

A method for foam fractionation that is independent of the aeration in the bioreactor systems has been presented and evaluated for application in lipopeptide production processes. The functionality of this external foam column for lipopeptide enrichment was demonstrated on the example of surfactin. The main obstacle was the severe foam formation during the aerated fermentation process, which challenged reproducibility. Therefore, differences in bacterial growth and surfactin production were observed between replicates, yet the average surfactin enrichment was comparable throughout the experiments. This demonstrated the robustness of the here applied method. However, due to the described challenges, the external foam column can only be conditionally recommended in the case of highly aerated surfactin production processes. The bioprocess should therefore be thoroughly assessed before applying ISPR. Moreover, a smart control system, that adapts to the process dynamics would be advantageous for future monitoring of foam fractionation applications. However, an application of an external foam column as shown here would be conceivable for non-foaming processes, since the aeration and foaming can be monitored independently from the bioreactor process, which is one of the highlights of this method.

## Data Availability

The raw data supporting the conclusions of this article will be made available by the authors, without undue reservation.
