# Supplementary material for: Evaluation of an external foam column for in situ product removal in aerated surfactin production processes
Source: Front Bioeng Biotechnol. 2023 Nov 6;11:1264787. doi: 10.3389/fbioe.2023.1264787 (PMC10657896; doi:10.3389/fbioe.2023.1264787)
Supplement: Supplementary file 1 [file DataSheet1.pdf]

Supplementary Material

Evaluation of an external foam column for *in situ* product removal in aerated surfactin production processes

Chantal Treinen, Linda Claassen, Mareen Hoffmann, Lars Lilge, Marius Henkel and Rudolf Hausmann*

*** Correspondence:**

Rudolf Hausmann

rudolf.hausmann@uni-hohenheim.de

Overview of selected online measurements

In the following an overview of online measurements during bioreactor cultivation are presented as supporting information. The figures are always structured in the same way and display four selected online measurements. Part **(A)** shows the dissolved oxygen pO_2 in [%]. The pO_2 was set to a minimum of 20%. Part **(B)** represents the reactor volume in [kg]. To determine the volume, the bioreactor was mounted on a scale and therefore the volume is given in [kg] and not in [L]. Outliers that appear in the form of dots are due to sampling or operations on the bioreactor, which caused deviations in the measured weight. Part **(C)** shows the aeration in [L/min]. An initial aeration of 1.4 L/min (0.07 vvm) was set. Part **(D)** shows the agitation rate as stirrer speed [rpm]. An initial stirrer speed of 300 rpm was set. Both the aeration and agitation were regulated automatically by the bioreactor system in dependence of the pO_2 if not stated otherwise in the specific figure.

Online measurements of reference processes

A

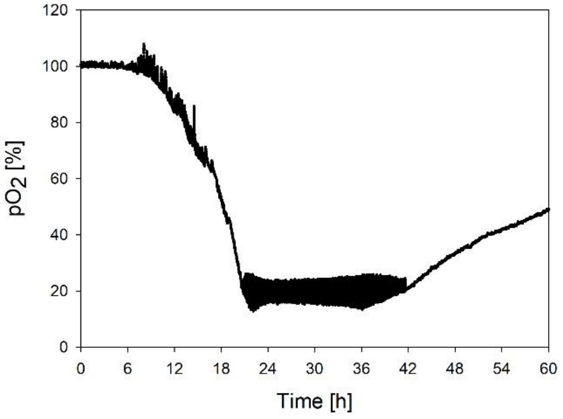

B

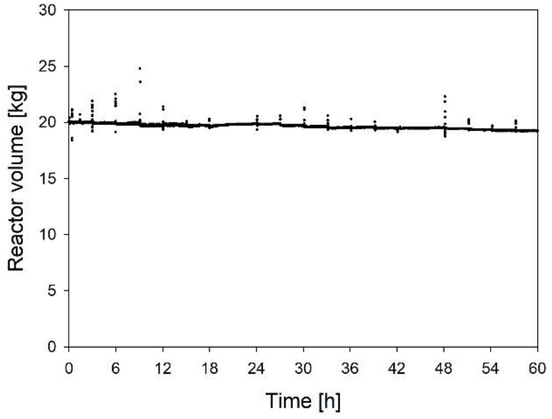

C

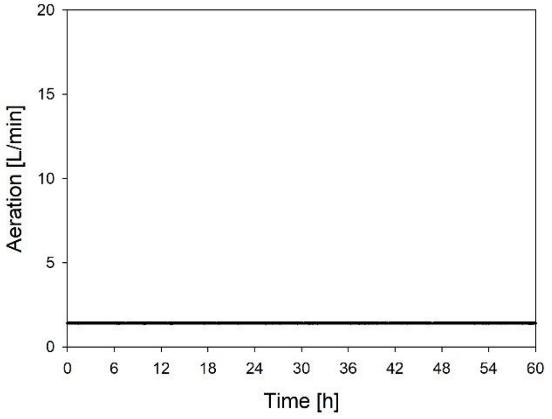

D

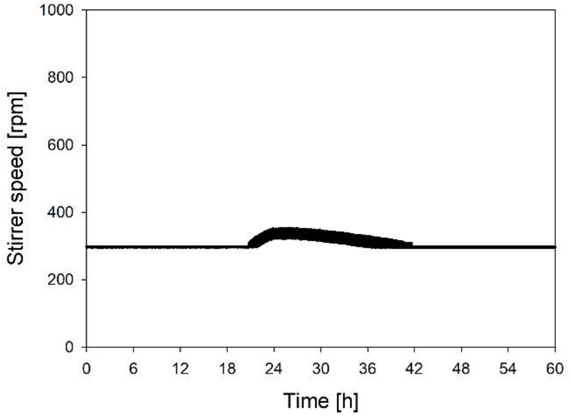

Supplementary Figure 1| Time-course of selected online measurements during the fermentation of *B. subtilis* JABs24. Reference process Replicate 1 with a cultivation time until t = 60 h.

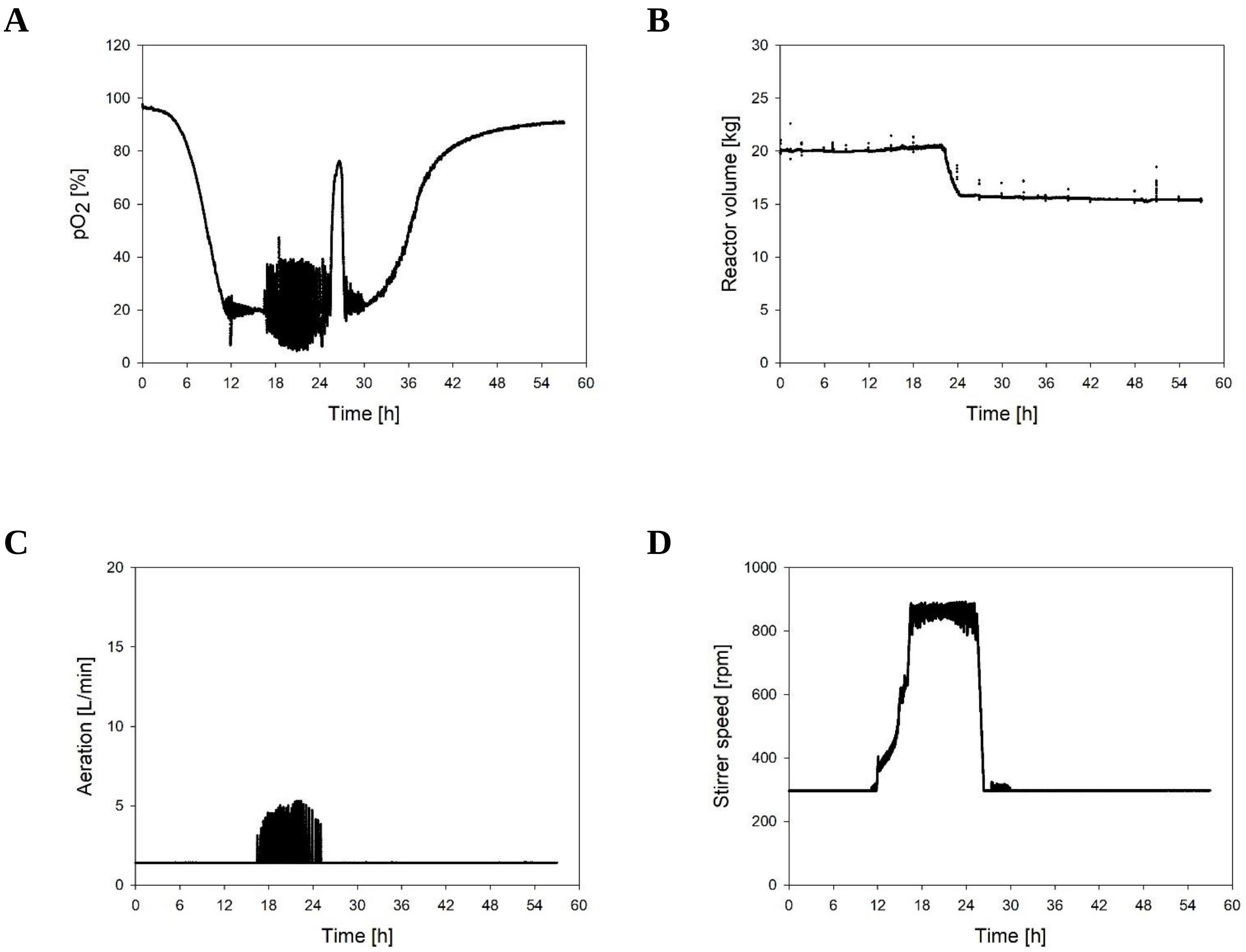

Supplementary Figure 2| Time-course of selected online measurements during the fermentation of *B. subtilis* JABs24. Reference process Replicate 2 with a cultivation time until t = 57 h. Antifoam agent was regularly refilled, as strong foam formation occurred. The timeline on the x-axis was approximated. Start and stop time during analysis were set based on the date and time the experiment was performed. However, deviations may occur during the transfer of the data from the bioreactor online data to the computer system. These typically amount to a few minutes to one hour and do not affect the overall trend.

Online measurements of foam column process

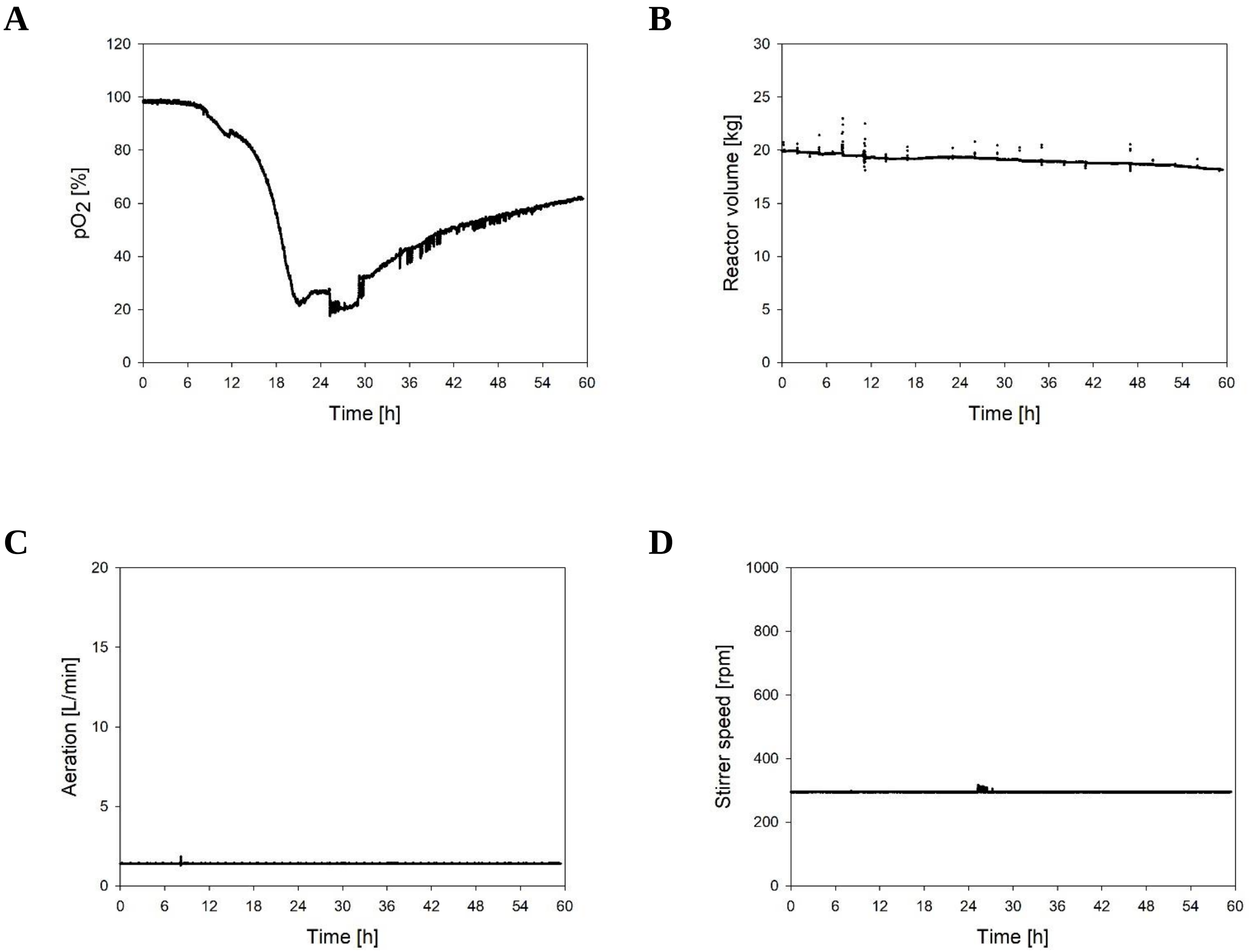

Supplementary Figure 3| Time-course of selected online measurements during the fermentation of *B. subtilis* JABs24. Foam column process Replicate 1 with a cultivation time until t = 60 h.

A

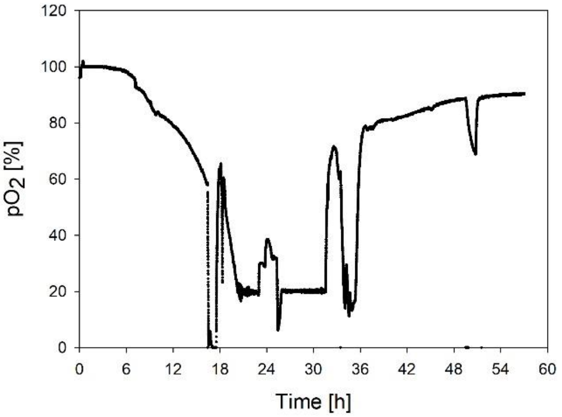

B

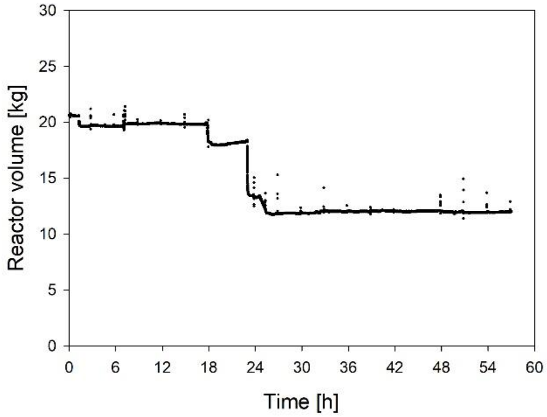

C

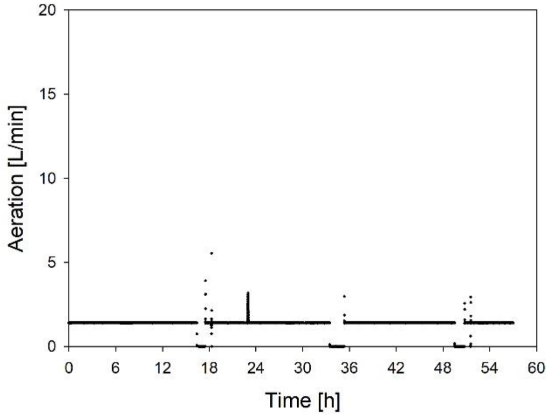

D

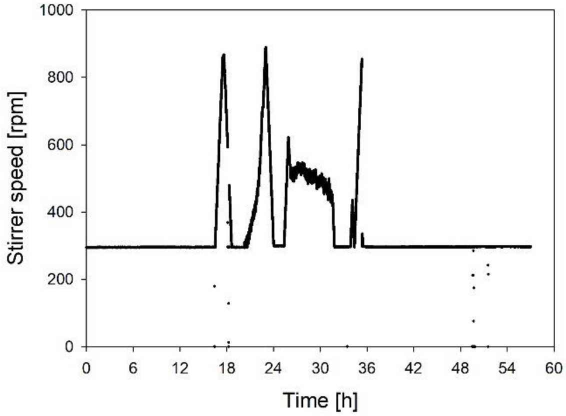

Supplementary Figure 4| Time-course of selected online measurements during the fermentation of *B. subtilis* JABs24. Foam column process Replicate 2 with a cultivation time until t = 57 h. Occasionally the pO_2 regulation failed and was restarted. Since the level of the pO_2 was sometimes to high (~ t = 18 h), the stirrer speed was set manually to 300 rpm until the pO_2 was decreased again. The timeline on the x-axis was approximated. Start and stop time during analysis were set based on the date and time the experiment was performed. However, deviations may occur during the transfer of the data from the bioreactor online data to the computer system. These typically amount to a few minutes to one hour and do not affect the overall trend.

Online measurements of negative control process

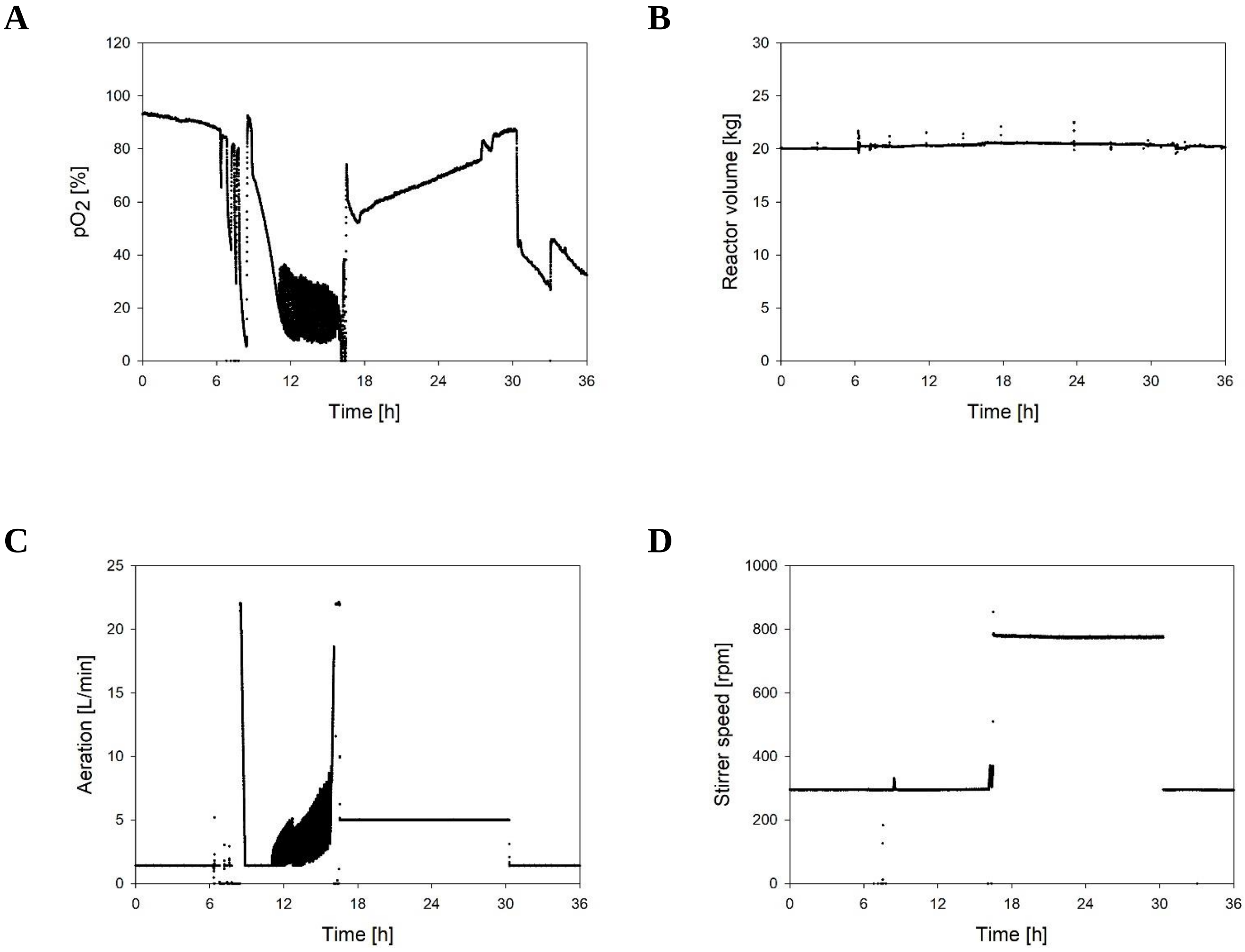

Supplementary Figure 5| Time-course of selected online measurements during the fermentation of *B. subtilis* 168 with a cultivation time until t = 36 h. Occasionally the pO_2 regulation failed and was restarted. To prevent the pO_2 to drop below 20%, the aeration and agitation was set manually overnight to 800 rpm and 5 L/min and was then again lowered to 300 rpm and 1.4 L/min towards the end of the cultivation.

Supplementary Table 1| Overview of results obtained by applying the foam column during a bioreactor process with *B. subtilis* JABs24 (Replicate 1). To calculate the absolute amount of surfactin $m_{surfactin}$, is was assumed that 1 g of foam equals 1 mL of foam. Mean values of $c_{surfactin}$ were used for the calculations of $m_{surfactin}$ with standard deviations typically ≤ 0.005 g.

	**Culture broth**	**Foam column - Foamate**						
**Time [h]**	**$c_{surfactin}$ [g/L]**	**Liquid flow [mL/min]**	**Gas flow [L/min]**	**Run-time [min]**	**Flow rate [$g_{foamate}$/min]**	**$c_{surfactin}$ [g/L]**	**Enrichment [-]**	**$m_{surfactin}$ [g]**
24	0.7 ± 0.1	20	3	20	1.91	2.7 ± 0.1	4.0	0.10
27	1.3 ± 0.3	20	3	20	0.99	3.0 ± 0.2	2.3	0.06
30	1.2 ± 0.2	20	3	20	0.74	3.7 ± 0.1	3.1	0.06
33	1.5 ± 0.0	20	3	20	0.86	3.6 ± 0.1	2.4	0.06
36	1.8 ± 0.0	20	3	20	0.84	4.2 ± 0.2	2.4	0.07
39	1.8 ± 0.1	20	3	20	1.02	3.9 ± 0.1	2.2	0.08
42	1.7 ± 0.0	20	3	20	0.85	3.7 ± 0.1	2.2	0.06
48	1.9 ± 0.0	20	3	23	0.88	5.2 ± 0.1	2.8	0.11
51	1.9 ± 0.0	20	3	20	0.80	6.4 ± 0.3	3.3	0.10
54	1.8 ± 0.1	20	3	18	0.93	5.3 ± 0.2	2.9	0.09
57	1.6 ± 0.0	20	3	19	0.84	3.8 ± 0.1	2.3	0.06
60	1.6 ± 0.1	20	3	20	0.95	3.6 ± 0.2	2.3	0.07

Supplementary Table 2| Overview of results obtained by applying the foam column during a bioreactor process with *B. subtilis* JABs24 (Replicate 2). To calculate the absolute amount of surfactin $m_{surfactin}$, is was assumed that 1 g of foam equals 1 mL of foam. Mean values of $c_{surfactin}$ were used for the calculations of $m_{surfactin}$ with standard deviations typically ≤ 0.01 g.

	**Culture broth**	**Foam column - Foamate**						
**Time** [h]	$c_{surfactin}$ [g/L]	**Liquid flow** [mL/min]	**Gas flow** [L/min]	**Run-time** [min]	**Flow rate** [$g_{foamate}$/min]	$c_{surfactin}$ [g/L]	**Enrichment** [-]	$m_{surfactin}$ [g]
30	2.0 ± 0.1	20	3	55	0.45	2.5 ± 0.4	1.3	0.06
33	2.3 ± 0.1	15	3	40	0.10	3.6 ± 0.8	1.5	0.01
36	2.4 ± 0.1	15	3	40	0.37	3.7 ± 0.7	1.5	0.05
39	2.5 ± 0.0	15	3	40	0.07	6.4 ± 0.1	2.6	0.02
42	2.6 ± 0.0	15	3	N/A	N/A	6.6 ± 0.5	2.6	N/A
48	2.6 ± 0.0	15	3	27	0.03	ND	ND	ND
51	2.3 ± 0.0	15	3	60	0.03	7.7 ± 0.4	3.3	0.01
54	2.5 ± 0.0	15	3	60	0.07	7.1 ± 0.5	2.8	0.03
57	2.5 ± 0.0	15	3	50	0.05	6.9 ± 0.4	2.7	0.02

ND = not determined, as the sample volume was not sufficient

Supplementary Table 3| Overview of conducted bioreactor processes with the foam column using *B. subtilis* JABs24 (not chronological).

Parameter	Replicate 1		Replicate 2		Replicate 3		Replicate 4		Replicate 5		Replicate 6		Replicate 7	
X_{max} [g/L] - Culture broth	7.4 ± 0.2	57 h	6.3 ± 0.2	30 h	4.7 ± 0.1	33 h	5.8 ± 0.2	51 h	17.3 ± 0.2*	27 h	3.8 ± 0.1	33 h	6.3 ± 0.0	31 h
P_{max} [g/L] - Culture broth	1.9 ± 0.0	51 h	2.6 ± 0.0	48 h	1.8 ± 0.2	30 h	3.7 ± 0.2	36 h	**	**	3.2 ± 0.2	51 h	2.7 ± 0.0	34.5 h
X_{max} [g/L] - Foamate	2.7 ± 0.0	51 h	5.7 ± 0.2	30 h	2.8 ± 0.0	30 h	3.2 ± 0.0	51 h	7.8 ± 0.2	30 h	3.7 ± 0.2	39 h	5.6 ± 0.2	31 h
P_{max} [g/L] - Foamate	6.4 ± 0.3	51 h	7.7 ± 0.4	51 h	1.8 ± 0.1	30 h	8.9**	27 h	1.3**	24 h	3.6 ± 0.0	30 h	5.7 ± 0.2	27 h
X $Enrichment_{max}$	0.5	60 h	2.4	54 h	0.8	24 h	0.7	57 h	1.1	30 h	1.3	39 h	1.1	34.5 h
P $Enrichment_{max}$	4.0	24 h	3.3	51 h	1.2	16.5 h	3.0	24 h	N/A	N/A	3.7	27 h	2.8	29 h
X $Enrichment_{mean}$	0.4 ± 0.1		1.8 ± 0.6		0.7 ± 0.1		0.6 ± 0.1		0.7 ± 0.2		0.9 ± 0.3		0.8 ± 0.2	
P $Enrichment_{mean}$	2.7 ± 0.5		2.3 ± 0.7		1.0 ± 0.1		3.3 ± 0.2		N/A		2.4 ± 1.0		2.2 ± 0.6	
Run-Time total	60 h		57 h		33 h		57 h		36 h		51 h		36 h	
Liquid flow	20 mL/min		15 – 20 mL/min		20 mL/min		10 – 20 mL/min		10 – 20 mL/min		N/A		10 – 15 mL/min	
Gas flow	3 L/min		3 L/min		3 – 9 L/min		3 L/min		3 – 6 L/min		N/A		6 – 7.5 L/min	

Supplemetary Table 3| continued

Parameter	Replicate 1	Replicate 2	Replicate 3	Replicate 4	Replicate 5	Replicate 6	Replicate 7
Non-return valve in recirculation	NO	YES	NO	YES	YES	YES	YES
Overfoaming	–	+	+	+	+	+	+
Comments	In Manuscript	In Manuscript; Occasional failure in pH and pO_2 regulation	< 3 kg of culture broth remaining; Occasional failure in pH, pO_2 and temperature regulation; Occasional pressure increase; Foaming out via recirculation tube	Not enough sample volume left for a comprehensive surfactin analysis	Occasional failure in pH and pO_2 regulation; Occasional pressure increase and clogged exhaust filter	Occasional failure in pH and pO_2 regulation; pO_2 regulation settings initially incorrect	Occasional failure in pH and pO_2 regulation; Very dense foam in bioreactor and foam column, which possibly affected functionality

*exceptionally high, other high-point at 9.2 ± 0.3 g/L after 24 h

**not all samples evaluated
